# Common Genetic Variation in the *SERPINF1* Locus Determines Overall Adiposity, Obesity-Related Insulin Resistance, and Circulating Leptin Levels

**DOI:** 10.1371/journal.pone.0034035

**Published:** 2012-03-23

**Authors:** Anja Böhm, Anna-Maria Ordelheide, Jürgen Machann, Martin Heni, Caroline Ketterer, Fausto Machicao, Fritz Schick, Norbert Stefan, Andreas Fritsche, Hans-Ulrich Häring, Harald Staiger

**Affiliations:** 1 Department of Internal Medicine, Division of Endocrinology, Diabetology, Angiology, Nephrology and Clinical Chemistry, Eberhard Karls University Tübingen, Tübingen, Germany; 2 Institute of Experimental Genetics, Group of Translational Diabetology, Helmholtz Centre Munich, German Research Center for Environmental Health, Munich, Germany; Member of the German Centre for Diabetes Research; 3 Institute for Diabetes Research and Metabolic Diseases of the Helmholtz Centre Munich at the University of Tübingen, Tübingen, Germany; Member of the German Centre for Diabetes Research; 4 Department of Diagnostic and Interventional Radiology, Section of Experimental Radiology, Eberhard Karls University Tübingen, Tübingen, Germany; 5 Department of Internal Medicine, Division of Nutritional and Preventive Medicine, Eberhard Karls University Tübingen, Tübingen, Germany; Universita Magna-Graecia di Catanzaro, Italy

## Abstract

**Objective:**

Pigment epithelium-derived factor (PEDF) belongs to the serpin family of peptidase inhibitors (serpin F1) and is among the most abundant glycoproteins secreted by adipocytes. In vitro and mouse in vivo data revealed PEDF as a candidate mediator of obesity-induced insulin resistance. Therefore, we assessed whether common genetic variation within the *SERPINF1* locus contributes to adipose tissue-related prediabetic phenotypes in humans.

**Subjects/Methods:**

A population of 1,974 White European individuals at increased risk for type 2 diabetes was characterized by an oral glucose tolerance test with glucose and insulin measurements (1,409 leptin measurements) and genotyped for five tagging SNPs covering 100% of common genetic variation (minor allele frequency ≥0.05) in the *SERPINF1* locus. In addition, a subgroup of 486 subjects underwent a hyperinsulinaemic-euglycaemic clamp and a subgroup of 340 magnetic resonance imaging (MRI) and spectroscopy (MRS).

**Results:**

After adjustment for gender and age and Bonferroni correction for the number of SNPs tested, SNP rs12603825 revealed significant association with MRI-derived total adipose tissue mass (p = 0.0094) and fasting leptin concentrations (p = 0.0035) as well as nominal associations with bioelectrical impedance-derived percentage of body fat (p = 0.0182) and clamp-derived insulin sensitivity (p = 0.0251). The association with insulin sensitivity was completely abolished by additional adjustment for body fat (p = 0.8). Moreover, the fat mass-increasing allele of SNP rs12603825 was significantly associated with elevated fasting PEDF concentrations (p = 0.0436), and the PEDF levels were robustly and positively associated with all body fat parameters measured and with fasting leptin concentrations (p<0.0001, all).

**Conclusion:**

In humans at increased risk for type 2 diabetes, a functional common genetic variant in the gene locus encoding PEDF contributes to overall body adiposity, obesity-related insulin resistance, and circulating leptin levels.

## Introduction

Pigment epithelium-derived factor (MIM ID *172860), also referred to as serpin F1 (human gene symbol: *SERPINF1*), belongs to the serpin family of peptidase inhibitors. Even though this serpin is unique in that its C-reactive loop is inactive and thus non-inhibitory [1], PEDF was reported to exert multiple effects in vitro and in mice in vivo including promotion of neuronal survival and differentiation and potent inhibition of angiogenesis [2]. Originally identified as a product of cultured human retinal pigment epithelium cells, PEDF is thought to play a central role in the development of the neural retina [3].

Recently, PEDF was identified as a bona fide adipokine by lectin chromatography of culture media conditioned by 3T3-L1 adipocytes and subsequent mass spectrometric analysis [4]. Based on peptide coverage in the mass spectra, PEDF accounted for ∼20% of full-length proteins and thus turned out to be among the most abundant glycoproteins secreted by 3T3-L1 adipocytes. Later on, studies on the secretome of human adipocytes using two-dimensional polyacrylamide gel electrophoresis of conditioned media followed by mass spectrometric analysis confirmed PEDF as one of the most abundant adipokines [5]. Furthermore, plasma PEDF concentrations were shown to be significantly elevated in human type 2 diabetes [6], [7] and the metabolic syndrome [8], [9]. In genetically and diet-induced obese mice, adipose tissue PEDF expression and plasma PEDF levels increased up to three-fold, whereas liver and skeletal muscle expressed only modest amounts of PEDF which did not increase upon obesity [4]. Caloric restriction of diet-induced obese mice resulted in a marked reduction in adipose tissue PEDF expression [4].

Based on experiments performed in vitro and in mice in vivo using recombinant PEDF, it could be demonstrated that this glycoprotein exerts direct short-term and indirect long-term effects on insulin-sensitive tissues, such as skeletal muscle, adipose tissue itself, and the liver [4], [5]. For the short term, PEDF is postulated to act via a cell surface receptor [10], [11] to activate c-Jun N-terminal kinase and extracellular signal-regulated kinases which in turn phosphorylate insulin receptor substrate 1 (IRS-1) at serine residues [4]. This is known to convert IRS-1 into an inhibitor of the insulin receptor tyrosine kinase. In consequence, PEDF treatment results in reduced in vivo insulin sensitivity, impaired insulin and glucose tolerance, increased hepatic glucose production, and decreased insulin-stimulated muscular glucose uptake in the absence of altered plasma insulin levels [4]. For the long term using miniosmotic pumps, PEDF was shown to increase adipose tissue lipolysis [4], an effect most probably mediated via adipose triglyceride lipase [12]. This promotes spill-over of free fatty acids to skeletal muscle and liver and ectopic lipid deposition in these tissues which promotes insulin resistance and reduced insulin-stimulated muscular glucose uptake [4]. All the acute and chronic effects could be blocked by a neutralizing anti-PEDF antibody [4].

Thus, PEDF represents a candidate mediator of obesity-induced insulin resistance. Its importance for human obesity and insulin resistance is however not well investigated. Therefore, we assessed in 1,974 White European individuals at increased risk for type 2 diabetes whether common genetic variation (minor allele frequency [MAF]≥0.05) within the *SERPINF1* locus contributes to adipose tissue-related prediabetic phenotypes, such as increased body adiposity, obesity-related insulin resistance, and elevated circulating levels of the adipokine leptin.

## Materials and Methods

### Ethics statement

Informed written consent to the study was obtained from all participants. The study adhered to the Declaration of Helsinki, and the study protocol was approved by the Ethics Committee of the Medical Faculty of the Eberhard Karls University Tübingen.

### Subjects

A study cohort of 1,974 White European individuals was recruited from the ongoing Tübingen family study for type 2 diabetes that currently encompasses more than 2,000 participants at increased risk for type 2 diabetes (non-diabetic individuals from Southern Germany with family history of type 2 diabetes or diagnosis of impaired fasting glycaemia) [13]. All participants underwent the standard procedures of the study protocol including assessment of medical history, smoking status and alcohol consumption habits, physical examination, routine blood tests, and an oral glucose tolerance test (OGTT). Selection of the present study cohort was based on the absence of newly diagnosed diabetes and the availability of complete sets of phenotype and genotype data. The participants were not taking any medication known to affect glucose tolerance or insulin secretion. From the overall cohort, a subgroup of 486 subjects voluntarily agreed to undergo a hyperinsulinaemic-euglycaemic clamp and a subgroup thereof (N = 340) additionally magnetic resonance imaging (MRI) and spectroscopy (MRS). The clinical characteristics of the overall cohort and the clamp and MRI/MRS subgroups are given in Table 1.

**Table 1:** Clinical characteristics of the study population.

A	Overall cohort (N = 1,974)		Clamp subgroup (N = 486)		MRI/MRS subgroup (N = 340)	
	Median	Interquartile range	Median	Interquartile range	Median	Interquartile range
Age (y)	39	21	40	21	47	16
BMI (kg/m^2^)	27.6	11.0	26.9	7.4	29.2	6.1
Body fat (%)	31.0	18.0	28.4	15.0	32.9	13.0
Waist circumference (cm)	93	24	91	20	98	18
Fasting leptin (ng/mL)*	16.6	31.8	12.4	20.9	20.3	26.9
Fasting glucose (mmol/L)	5.11	0.66	5.00	0.66	5.12	0.73
Glucose 120 min OGTT (mmol/L)	6.21	2.11	6.06	2.07	6.67	1.97
Fasting insulin (pmol/L)	50	52	40	35	50	46
HOMA-IR (mmol*mU*L^−2^)	1.92	2.17	1.53	1.42	1.93	1.84
ISI OGTT (*10^15^ L^2^*mol^−2^)	12.6	13.3	15.6	13.8	11.4	9.1
ISI clamp (*10^6^ L*kg^−1^*min^−1^)	-	-	0.072	0.060	0.058	0.039
Total adipose tissue (% BW)	-	-	-	-	30.2	14.0
Visceral adipose tissue (% BW)	-	-	-	-	3.16	2.46
Intrahepatic lipids (%)	-	-	-	-	3.47	6.34
Fasting PEDF (µg/ml)	-	-	-	-	7.85	2.72

B	Overall cohort (N = 1,974)						Clamp subgroup (N = 486)						MRI/MRS subgroup (N = 340)					
	Gender		Glucose tolerance status				Gender		Glucose tolerance status				Gender		Glucose tolerance status			
	women	men	NGT	IFG	IGT	IFG & IGT	women	men	NGT	IFG	IGT	IFG & IGT	women	men	NGT	IFG	IGT	IFG & IGT
Number	1,305	669	1,390	223	194	167	263	223	368	36	49	33	208	132	212	44	45	39
Proportion (%)	66.1	33.9	70.4	11.3	9.8	8.5	54.1	45.9	75.7	7.4	10.1	6.8	61.2	38.8	62.4	12.9	13.2	11.5

AUC – area under the curve; BMI – body mass index; BW – body weight; C-Pep – C-peptide; Glc – glucose; HOMA-IR – homeostasis model assessment of insulin resistance; Ins – insulin; ISI – insulin sensitivity index; MRI – magnetic resonance imaging; MRS – magnetic resonance spectroscopy; OGTT – oral glucose tolerance test; *available from 1,409 participants.

IFG – impaired fasting glycaemia; IGT – impaired glucose tolerance; NGT – normal glucose tolerance.

### OGTT

A standardized 75-g OGTT was performed after a 10-h overnight fast, and venous blood samples were drawn at time-points 0, 30, 60, 90, and 120 min for the determination of plasma glucose and insulin concentrations [13].

### Hyperinsulinaemic-euglycaemic clamp

After a 10-h overnight fast and a 60-min baseline period, subjects received a priming dose of insulin followed by an infusion (40 mU/m^2^) of short-acting human insulin for 120 min. A variable infusion of 20% glucose was started to maintain the plasma glucose concentration at 5.5 mmol/L. Blood samples for the measurement of plasma glucose were obtained at 5-min intervals throughout the clamp, plasma insulin levels were measured at baseline and in the steady state of the clamp [13].

### Measurements of body fat content and distribution

Body mass index (BMI) was calculated as weight divided by height squared (kg/m^2^). Waist circumference (in cm) was measured in the upright position at the midpoint between the lateral iliac crest and the lowest rib. The percentage of body fat was measured by bioelectrical impedance (BIA-101, RJL systems, Detroit, MI, USA). In addition, total and visceral fat contents (in % of body weight) were determined by MRI with an axial T1-weighted fast-spin echo technique with a 1.5-T whole-body imager (Magnetom Sonata, Siemens Medical Solutions, München, Germany), as described earlier [14]. The intrahepatic lipid content (in % of signal) was determined by localized STEAM ^1^H-MRS (TR = 4 s, TE = 10 ms, 32 scans) in the 7^th^ segment of the liver, as formerly reported [15].

### Laboratory measurements

Plasma glucose (in mmol/L) was determined using a bedside glucose analyzer (glucose oxidase method, Yellow Springs Instruments, Yellow Springs, OH, USA). Plasma insulin concentrations (in pmol/L) were measured by a commercial chemiluminescence assay for ADVIA Centaur (Siemens Medical Solutions, Fernwald, Germany) according to the manufacturer's instructions. Fasting plasma leptin (in ng/mL) and PEDF (in µg/ml) concentrations were determined by enzyme-linked immunosorbent assays (Linco Research, St. Charles, MO, and BioVendor, Heidelberg, Germany, respectively).

### Selection of tagging single nucleotide polymorphisms (SNPs)

**Figure 1:**
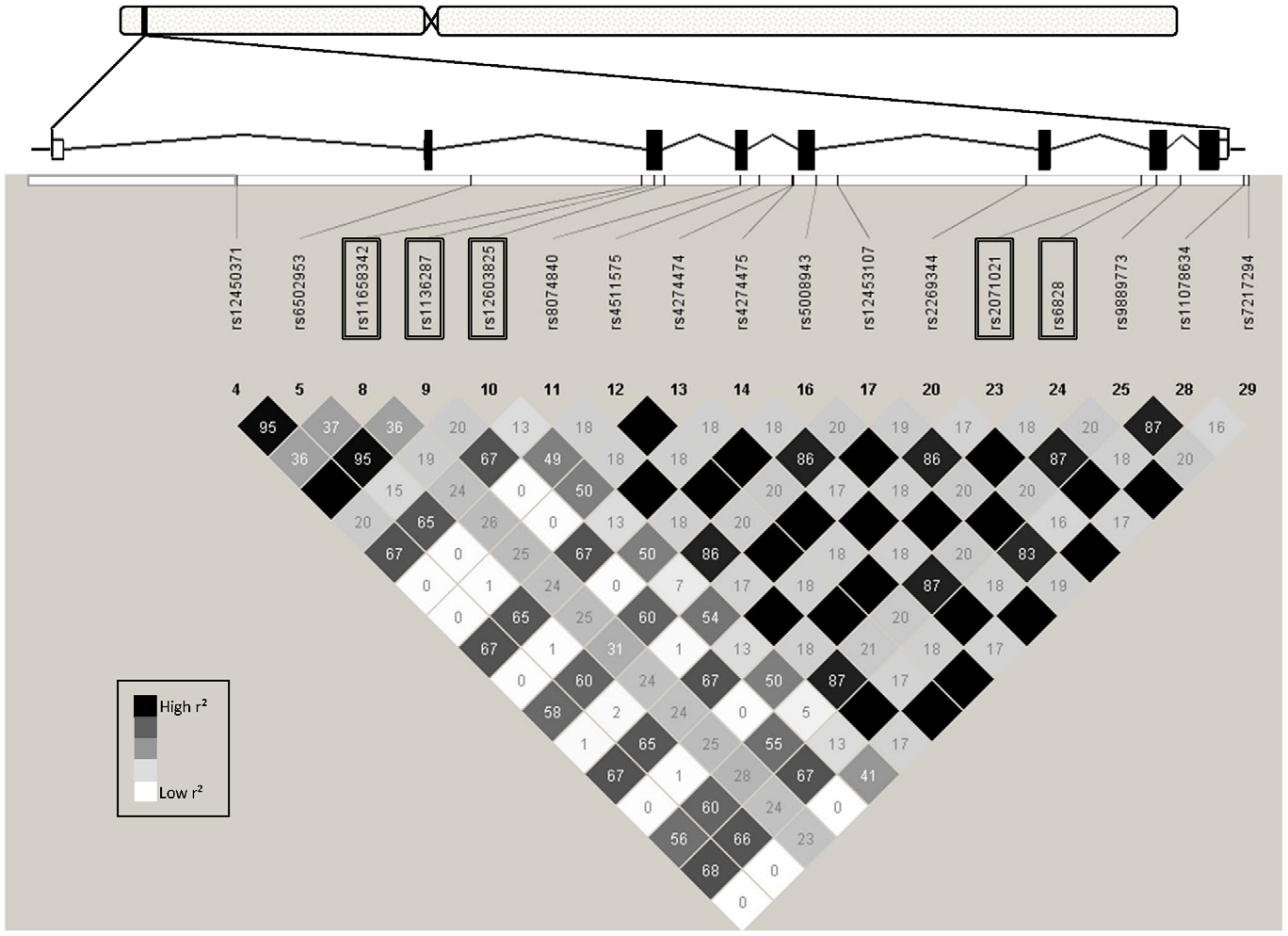
Genomic region of human chromosome 17p13.3 harbouring the *SERPINF1* gene and HapMap linkage disequilibrium data of common (minor allele frequency ≥0.05) informative SNPs within this region. The *SERPINF1* gene consists of 8 exons and 7 introns and spans 15.6 kb from nucleotide position 1,612,009 to nucleotide position 1,627,617. The analyzed region additionally included 4 kb of the 5′-flanking region and 1.5 kb of the 3′-flanking region. This genomic region did not overlap with other known gene loci. The locations of the five tagging SNPs (highlighted by boxes) are indicated. Linkage disequilibrium data, i.e., r^2^-values only, are presented as numbers in the diamonds (100 = 1.00) and different diamond shadings (white – low linkage; black – high linkage; grey – in between).

Based on the publicly available phase III data of the International HapMap Project derived from the CEU population of Utah residents with ancestry from Northern and Western Europe (release #28 August 2010, http://hapmap.ncbi.nlm.nih.gov/index.html.en), we screened *in silico* the complete *SERPINF1* gene spanning 15.6 kb (8 exons, 7 introns, located on human chromosome 17p13.3) as well as 4 and 1.5 kb of its 5′- and 3′-flanking regions, respectively (Figure 1). The *SERPINF1* locus is flanked 6.5 kb upstream by the *SERPINF2* gene (on the same DNA strand) and 1.5 kb downstream by the *SMYD4* gene (on the reverse DNA strand), but no linkage blocks within the screened *SERPINF1* locus region were found to overlap with these neighbouring genes. Within the *SERPINF1* locus, 30 informative HapMap SNPs were present with 17 displaying MAFs≥0.05. The HapMap linkage disequilibrium (r^2^) data of the 17 common SNPs are schematically presented in Figure 1. Among these, five SNPs were selected tagging all the other common SNPs within the locus with an r^2^>0.8 (100% coverage) based on Tagger analysis using Haploview software (http://www.broadinstitute.org/scientific-community/science/programs/medical-and-population-genetics/haploview/haploview). As depicted in Figure 1, these five tagging SNPs were rs11658342 (G/A) in intron 2, rs1136287 (T/C) in exon 3 encoding the amino acid exchange Met72Thr, rs12603825 (G/A) in intron 3, rs2071021 (A/G) in intron 6, and rs6828 (C/T) in exon 7 encoding the synonymous variation Tyr321Tyr.

### Genotyping

DNA was isolated from whole blood using a commercial DNA isolation kit (NucleoSpin, Macherey & Nagel, Düren, Germany). The five *SERPINF1* SNPs were genotyped using the Sequenom massARRAY system with iPLEX software (Sequenom, Hamburg, Germany). The genotyping success rates were ≥99.7%. The Sequenom results were validated by bidirectional sequencing in 50 randomly selected subjects, and both methods gave 100% identical results.

### Calculations

Homeostasis model assessment of insulin resistance (HOMA-IR) was calculated as {c(glucose[mmol/L])_0_ · c(insulin[mU/L])_0_}/22.5 with c = concentration [16]. The insulin sensitivity index derived from the OGTT (ISI OGTT) was estimated as proposed by Matsuda and DeFronzo [17]: 10,000/{c(glucose[mmol/L])_0_ · c(insulin[pmol/L])_0_ · c(glucose[mmol/L])_mean_ · c(insulin[pmol/L])_mean_}^½^. The insulin sensitivity index derived from the hyperinsulinaemic-euglycaemic clamp (ISI clamp) was calculated as glucose infusion rate necessary to maintain euglycaemia during the last 60 min (steady state) of the clamp (in µmol · kg^−1^ · min^−1^) divided by the steady-state insulin concentration (in pmol/L).

### Statistical analyses

Hardy-Weinberg equilibrium was tested using χ^2^ test. Linkage disequilibrium (D′, r^2^) between the tagging SNPs was analyzed using the JLIN programme provided by the Western Australian Institute for Medical Research (http://www.genepi.org.au/jlin). All continuous variables not normally distributed were log*_e_*-transformed prior to linear regression analysis. Multiple linear regression analysis was performed using the least-squares method. In the regression models, the trait of interest (measure of body fat content or distribution, plasma PEDF concentration, glycaemia, or insulin sensitivity) was chosen as dependent variable, the SNP genotype (in the additive or dominant inheritance model) as independent variable, and gender, age, and – when testing glycaemia or insulin sensitivity – percentage of body fat as confounding variables. Based on screening five non-linked tagging SNPs in parallel, a p-value<0.0102 was considered statistically significant according to Bonferroni correction for multiple comparisons. We did not correct (i) for the tested traits of interest since these were not independent and (ii) for the two inheritance models applied because associations were considered reliable only when observable in both models (at least significant in one model and nominal in the other). In all subsequent analyses addressing exclusively the effects of SNP rs12603825 in more detail, a p-value<0.05 was considered statistically significant. To perform these analyses, the statistical software package JMP 8.0 (SAS Institute, Cary, NC, USA) was used. In the dominant inheritance model, our overall study cohort was sufficiently powered (1-β≥0.8) to detect, for the five tagging SNPs, (unadjusted) effect sizes of Cohen'd ∼0.12, the clamp subgroup was sufficiently powered to detect effect sizes of ∼0.26, and the MRI/MRS subgroup to detect effect sizes of ∼0.30 (α<0.05; Figure S1) with Cohen's d-values of 0.2, 0.5, and 0.8 representing by convention small, medium, and large effect sizes, respectively. Power calculations were performed using G*power 3.0 software available at http://www.psycho.uni-duesseldorf.de/aap/projects/gpower/.

## Results

### Characteristics of the study participants

The overall study population consisted of 1,974 non-diabetic, relatively young (median age 39 y), and moderately overweight (median BMI 27.6 kg/m^2^) White Europeans. Two thirds of the subjects were women, one third men. About 70% of the subjects were normal glucose tolerant, 30% prediabetic: 11% had isolated impaired fasting glycaemia, 10% isolated impaired glucose tolerance, and 8.5% both disturbances of glucose homeostasis. The clinical characteristics of the study participants are given in Table 1.

### Genotyping results

**Figure 2:**
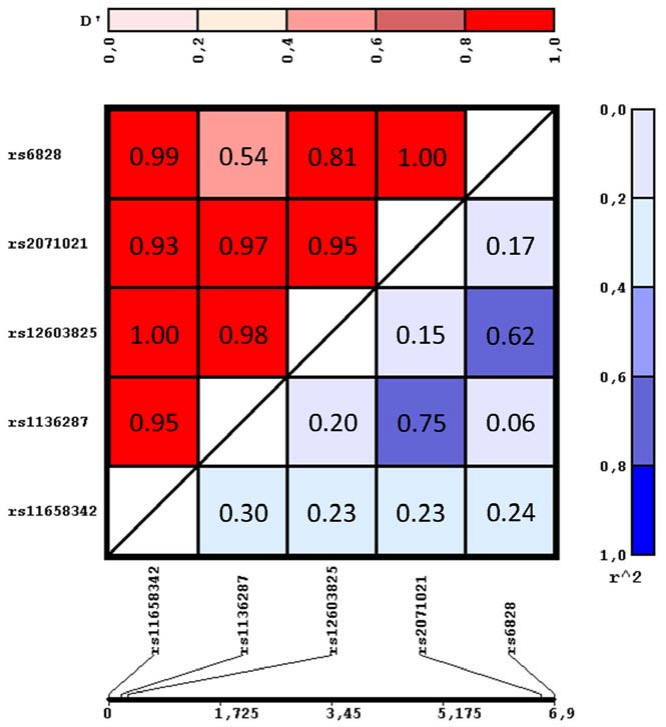
Linkage disequilibrium data of the five tagging SNPs derived from the overall cohort (N = 1,974). D′-values are marked by red shadings (scale given at the top), r^2^-values by blue shadings (scale given to the right). The distances (in kb) between the tagging SNPs are given at the bottom of the figure.

The 1,974 participants were genotyped for the five tagging SNPs of the *SERPINF1* gene locus (Figure 1) with genotyping success rates ≥99.7%. All SNPs were in Hardy-Weinberg equilibrium (p≥0.14, all). The observed MAFs ranged from 0.27 to 0.38 and were pretty similar to those reported by the HapMap consortium for the CEU population (Table S1). The genetic linkage between the tagging SNPs ranged from ‘weak’ (D′ = 0.54, r^2^ = 0.06) to ‘moderate’ (D′ = 0.97, r^2^ = 0.75, Figure 2).

### SNP associations with body fat content and distribution

**Figure 3:**
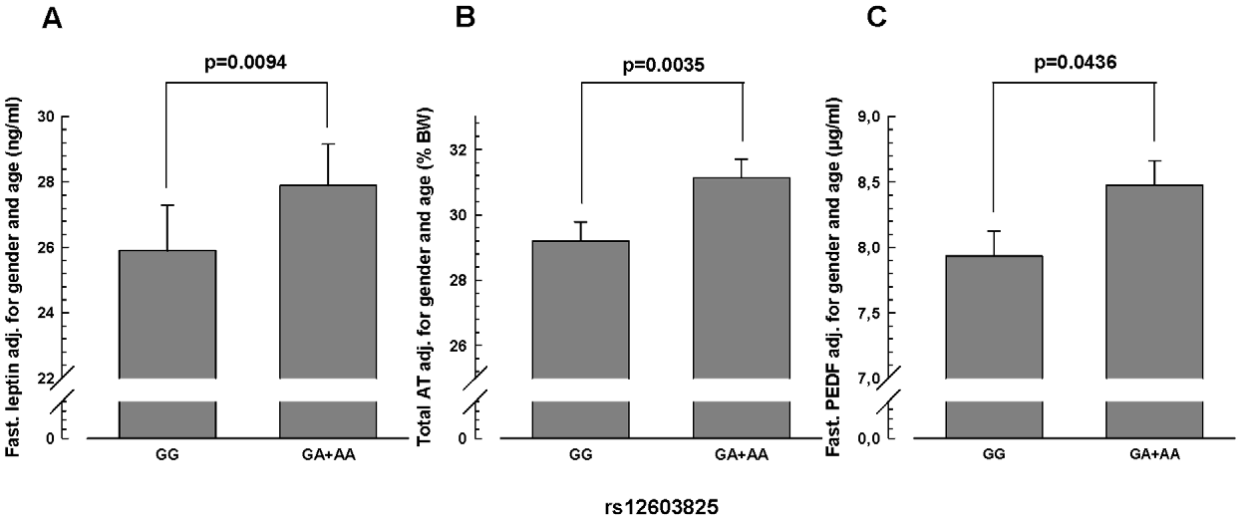
Association of SNP rs12603825 with fasting plasma leptin concentration (A), total adipose tissue mass (B), and fasting plasma PEDF concentration (C). Adjusted data (means +SE) of the dominant inheritance model (GG versus GA+AA) are presented. Total adipose tissue (AT) mass was measured by magnetic resonance imaging (N = 340), plasma leptin (N = 1,409) and PEDF (N = 340) by enzyme-linked immunosorbent assays (see Materials and Methods).

All SNP associations with body fat measures were studied after adjustment for gender and age. In the overall cohort, the minor A-allele of SNP rs12603825 was nominally associated with an elevated percentage of body fat (measured by bioelectrical impedance) in the dominant inheritance model (p = 0.0182; Table 2). In the additive model, only a trend was visible (p = 0.07; Table 2). However, this allele's significant association in the dominant model (p = 0.0035) and nominal association in the additive model (p = 0.0155) with increased fasting leptin levels supports this SNP's impact on overall adiposity (see Table 2 for raw data and Figure 3A for adjusted data). The adjusted effect size for this SNP's effect on plasma leptin was +1.54 ng/mL (+5.5% relative to GG) per risk allele. Inclusion of bioelectrical impedance-derived percentage of body fat in the multiple regression analysis abolished this SNP's association with plasma leptin (p = 0.2, additive and dominant model) showing that this effect on leptin is mediated by fat mass. In addition, the A-allele of rs12603825 revealed significant association in the dominant model (p = 0.0094) and nominal association in the additive model (p = 0.0250) with increased total adipose tissue mass, as measured by MRI (see Table 2 for raw data and Figure 3B for adjusted data). The adjusted effect size of SNP rs12603825 was +1.22% of body weight (+4.1% relative to GG) per risk allele.

**Table 2:** Associations of *SERPINF1* SNPs with parameters of body fat content and distribution (overall cohort and MRI/MRS subgroup).

	Genotype	N overall cohort	BMI (kg/m^2^)	Body fat (%)	Waist circum-ference (cm)	Fasting leptin (ng/mL)*	N MRI/MRS subgroup	Total AT(% BW)	Visceral AT(% BW)	Intrahepatic lipids (%)
rs11658342	GG	784	29.8±9.0	32.1±12.1	95±19	29.0±33.3	144	30.2±9.3	3.51±1.79	6.16±6.75
	GA	909	30.4±9.3	33.1±11.9	96±19	29.2±32.9	151	31.0±9.1	3.21±1.70	5.81±6.08
	AA	278	30.5±10.3	33.0±13.0	96±20	28.7±35.7	44	30.0±8.9	3.24±1.71	5.18±6.69
p_add_/p_dom_	-	-	0.4/0.3	0.6/0.3	0.3/0.2	0.3/0.4	-	0.4/0.5	0.7/0.7	0.2/0.5
rs1136287	TT	788	30.5±9.2	33.7±12.2	96±19	30.4±33.8	139	31.5±8.7	3.33±1.77	6.03±6.70
	TC	953	30.0±9.3	32.1±11.9	96±19	28.0±32.6	166	30.0±9.3	3.34±1.72	5.64±6.32
	CC	233	29.9±9.8	31.8±12.6	95±19	29.1±35.3	35	29.2±10.2	3.32±1.82	6.45±6.00
p_add_/p_dom_	-	-	0.3/0.2	**0.0298**/0.06	0.2/0.4	0.2/0.09	-	0.2/0.2	0.5/0.4	0.6/1.0
rs12603825	GG	1,039	30.1±9.7	32.2±12.4	95±19	27.8±33.4	168	29.8±9.6	3.22±1.71	5.69±6.30
	GA	784	30.4±8.9	33.4±11.8	96±19	31.0±34.1	138	31.3±8.4	3.40±1.76	5.80±6.17
	AA	144	30.0±8.6	32.6±11.8	95±17	28.2±30.4	32	31.6±10.0	3.60±1.87	7.40±8.22
p_add_/p_dom_	-	-	0.5/0.3	0.07/**0.0182**	0.7/0.6	**0.0155**/**0.0035**	**-**	**0.0250**/**0.0094**	0.2/0.2	0.4/0.4
rs2071021	AA	937	30.5±9.3	33.5±12.3	96±19	30.6±34.5	162	31.2±9.2	3.30±1.73	5.81±6.46
	AG	861	29.9±9.3	31.9±11.9	95±19	27.3±32.0	150	30.0±9.2	3.39±1.73	6.13±6.78
	GG	176	29.9±9.6	32.2±12.3	95±19	29.6±34.5	28	29.4±8.7	3.29±1.97	4.95±3.96
p_add_/p_dom_	-	-	0.2/0.1	0.1/0.1	0.2/0.3	0.2/0.06	**-**	0.4/0.3	0.7/0.8	0.6/0.6
rs6828	CC	1,005	30.1±9.5	32.4±12.3	96±19	27.9±33.4	167	29.7±9.0	3.32±1.78	5.79±6.52
	CT	806	30.3±9.2	32.9±11.9	96±20	29.9±33.3	137	31.5±9.0	3.26±1.67	5.80±5.95
	TT	161	30.5±8.9	33.5±12.5	95±17	31.8±34.1	36	30.8±10.3	3.69±1.86	6.62±7.80
p_add_/p_dom_	-	-	0.4/0.4	0.3/0.3	0.7/0.7	0.05/0.08	-	0.1/0.05	0.3/0.2	0.6/0.5

Data are shown as unadjusted raw data (means ±SD). Prior to statistical analysis, all parameters were adjusted for gender and age. p_add_ – p-value, additive inheritance model; p_dom_ – p-value, dominant inheritance model; nominal associations are marked by bold fonts, significant associations by bold fonts and underlining. AT – adipose tissue; BMI – body mass index; BW – body weight; MRI – magnetic resonance imaging; MRS – magnetic resonance spectroscopy; SNP – single nucleotide polymorphism; *available from 1,409 participants.

The *SERPINF1* SNP rs12603825 was not significantly associated with increased BMI, waist circumference, visceral adipose tissue mass, or intrahepatic lipids (p≥0.2, all; Table 2).

The other SNPs did not show any reliable association with measures of body fat content and distribution (Table 2).

### SNP associations with glycaemia and insulin sensitivity

All SNP associations with measures of glycaemia and insulin sensitivity were studied after adjustment for gender, age, and bioelectrical impedance-derived percentage of body fat. None of the SNPs revealed any significant effect on glycaemia or insulin sensitivity (p≥0.03, all; Table S2).

To see whether SNP rs12603825 exerts an effect on insulin sensitivity via its effect on body adiposity, we excluded percentage of body fat as a confounder from the multiple regression analysis. In the dominant inheritance model, the minor A-allele of SNP rs12603825 was significantly associated with reduced clamp-derived insulin sensitivity (p = 0.0251), and this association was completely abolished after inclusion of percentage of body fat in the analysis (p = 0.8; Table S2). This provides evidence for an association of this SNP with insulin resistance via promotion of body adiposity.

### Functionality of SNP rs12603825

SNP rs12603825 is located in intron 3 of the *SERPINF1* gene. Thus, it is supposed to affect *SERPINF1* transcription rather than the function or stability of PEDF. To address whether this SNP is functional, we first analysed in silico whether the SNP alters a transcription factor binding site. Using publically available prediction software (TFSEARCH at http://www.cbrc.jp/research/db/TFSEARCH.html and TESS at http://www.cbil.upenn.edu/cgi-bin/tess/tess?RQ=WELCOME), we analysed a DNA region from 20 bp upstream to 20 bp downstream of the SNP for putative transcription factor binding sites. However, no binding sites for mammalian transcription factors could be identified to be directly affected by the SNP.

Next, we assessed the in vivo functionality of SNP rs12603825 by testing whether the SNP influences the fasting plasma PEDF levels that were measured in the MRI/MRS subgroup. After adjustment for gender and age, the body fat-increasing A-allele of SNP rs12603825 tended to associate with higher fasting plasma PEDF in the additive inheritance model (p<0.1) and was significantly associated with increased plasma PEDF concentrations in the dominant model (p = 0.0436; Figure 3C).

Notably, the plasma PEDF concentrations adjusted for gender and age were very robustly and positively associated with BMI (β = 0.68±0.10, p<0.0001), bioelectrical impedance-derived percentage of body fat (β = 0.75±0.14, p<0.0001), MRI-derived total (β = 0.83±0.16, p<0.0001) and visceral (β = 0.12±0.03, p<0.0001) adipose tissue mass, MRS-derived intrahepatic lipids (β = 0.89±0.13, p<0.0001), and with fasting plasma leptin concentrations (β = 2.50±0.56, p<0.0001).

## Discussion

In this genetic study, we demonstrate in vivo functionality of the common *SERPINF1* variant rs12603825 and its influence on overall adiposity with the minor A-allele representing the plasma PEDF- and body fat-elevating risk allele. Why we could not detect an impact of this SNP on BMI could have several reasons. One conceivable explanation could be the relatively low age of the subjects examined (median age 39 y), as it is well-known that in young, physically active subjects BMI rather reflects muscle mass than fat mass. Another reason could be this SNP's modest effect size on body adiposity of ∼8% (AA versus GG) that is presumably too small to be translated into significant changes in BMI at least in our cohort of limited sample size. Interrogation of publically available genome-wide analyses from the GIANT consortium (http://www.broadinstitute.org/collaboration/giant/index.php/GIANT_consortium_data_files) again failed to reveal a significant association of SNP rs12603825 with BMI in approximately 250,000 subjects (p = 0.9). The lack of association in this large sample could be due to confounders, such as ethnicity, environment, prediabetic status, and study methods, that were not accounted for in this study [18]. Thus, replication of our results in larger, very well phenotyped and controlled study cohorts could help shed further light on this issue. Nevertheless, our finding, confirmed by the use of different measures of body adiposity (MRI-derived total adipose tissue mass, bioelectrical impedance-derived percentage of body fat, and plasma leptin concentration), is in line with earlier studies showing positive associations of circulating PEDF with obesity in rodents [4] and humans [9], [19], [20], with human type 2 diabetes [6], [7] and the metabolic syndrome [8], [9].

Whether the intronic variant rs12603825 affects the function or the expression of the gene product PEDF is currently unclear, but altered *SERPINF1* expression via modified transcription factor binding to the DNA sequence affected by the nucleotide exchange would be a conceivable explanation. Using publically available software tools, we were unable to identify predicted transcription factor binding sites directly altered by the SNP. Since these tools are however of limited precision, future in vitro studies are needed to clarify whether the SNP influences the binding of transcription factors to this intronic DNA sequence. According to the direction of the effects (increased body adiposity and reduced insulin sensitivity in A-allele carriers) and the supposed biological function of PEDF, one might postulate that the A-allele represents a gain-of-function nucleotide exchange.

Even though it is well-described for many adipokines (leptin, interleukin-6, plasminogen activator inhibitor 1, etc.) that increased fat mass results in elevated expression and secretion of the adipokine, it is unknown how primary alterations in PEDF expression/secretion, e.g., due to non-coding genetic variation in *SERPINF1*, affects body fat mass. In vitro and in mice in vivo, PEDF was reported to increase lipolysis in an auto-/paracrine way [4], [12], a property that could explain altered fat mass in carriers of SNP rs12603825. However, we were not able to show any association of SNP rs12603825 with lipolysis-derived free fatty acid or glycerol levels in the fasting state or during the OGTT (data not shown). Thus, the mechanism underlying the observed association remains unclear and awaits further molecular investigations.

Our finding that the A-allele of SNP rs12603825 was (nominally) associated with reduced clamp-derived insulin sensitivity (adjusted effect size −11.7%, AA versus GG) and that this association was completely abolished upon adjustment for body fat is consistent with our working hypothesis which postulated a role of PEDF in obesity-related insulin resistance, as derived from in vitro and mouse studies [4], [5]. Moreover, this result is in line with recently published human data reporting association of increased plasma PEDF concentrations with insulin resistance [19]–[21]. Since our finding did not reach the level of significance, replication studies in larger cohorts (or consortia) with hyperinsulinaemic-euglycaemic clamp data are required to confirm this SNP effect.

In conclusion, our study revealed that a functional common genetic variant in the gene locus encoding PEDF contributes to overall body adiposity, obesity-related insulin resistance, and circulating leptin levels in humans at increased risk for type 2 diabetes. This may support a role of PEDF in the pathogenesis of human obesity and obesity-related disorders, such as insulin resistance and type 2 diabetes.

## Supporting Information

Figure S1 **Power statistics of the five tagging SNPs in the overall cohort, the clamp subgroup, and the MRI/MRS subgroup.** The statistical power (1-β) and the smallest, still detectable effect size (Cohen's d) are plotted. By convention, Cohen's d-values of 0.2, 0.5, and 0.8 represent small, medium, and large effect sizes, respectively. Due to the narrow range of minor allele frequencies (0.27–0.38), the five SNPs form close bundles of curves in the three groups.
(TIF)

Table S1 **Minor allele frequencies of the five** ***SERPINF1*** **tagging SNPs observed in the overall cohort compared to HapMap CEU data.** MAF – minor allele frequency; SNP – single nucleotide polymorphism
(DOCX)

Table S2 **Associations of SERPINF1 SNPs with glycaemia and insulin sensitivity (overall cohort and clamp subgroup).** Data are shown as unadjusted raw data (means ±SD). Prior to statistical analysis, all parameters were adjusted for gender, age, and bioelectrical impedance-derived percentage of body fat. padd – p-value, additive inheritance model; pdom – p-value, dominant inheritance model; nominal associations are marked by bold fonts. HOMA-IR – homeostasis model assessment of insulin resistance; ISI – insulin sensitivity index; OGTT – oral glucose tolerance test; SNP – single nucleotide polymorphism
(DOCX)
