# Supplementary material for: Common Genetic Variation in the SERPINF1 Locus Determines Overall Adiposity, Obesity-Related Insulin Resistance, and Circulating Leptin Levels
Source: PLoS One. 2012 Mar 23;7(3):e34035. doi: 10.1371/journal.pone.0034035 (PMC3311576; doi:10.1371/journal.pone.0034035)
Supplement: Table S1 — Minor allele frequencies of the five SERPINF1 tagging SNPs observed in the overall cohort compared to HapMap CEU data. MAF – minor allele frequency; SNP – single nucleotide polymorphism (DOCX) [file pone.0034035.s002.docx]

**Table S1. Minor allele frequencies of the five *SERPINF1* tagging SNPs observed in the overall cohort compared to HapMap CEU data**

| SNP | MAF overall cohort | MAF HapMap CEU |
| --- | --- | --- |
| rs11658342 | 0.38 | 0.41 |
| rs1136287 | 0.35 | 0.36 |
| rs12603825 | 0.27 | 0.23 |
| rs2071021 | 0.30 | 0.28 |
| rs6828 | 0.28 | 0.29 |

MAF – minor allele frequency; SNP – single nucleotide polymorphism
