# Supplementary material for: Common Genetic Variation in the SERPINF1 Locus Determines Overall Adiposity, Obesity-Related Insulin Resistance, and Circulating Leptin Levels
Source: PLoS One. 2012 Mar 23;7(3):e34035. doi: 10.1371/journal.pone.0034035 (PMC3311576; doi:10.1371/journal.pone.0034035)
Supplement: Table S2 — Associations of SERPINF1 SNPs with glycaemia and insulin sensitivity (overall cohort and clamp subgroup). Data are shown as unadjusted raw data (means ±SD). Prior to statistical analysis, all parameters were adjusted for gender, age, and bioelectrical impedance-derived percentage of body fat. padd – p-value, additive inheritance model; pdom – p-value, dominant inheritance model; nominal associations are marked by bold fonts. HOMA-IR – homeostasis model assessment of insulin resistance; ISI – insulin sensitivity index; OGTT – oral glucose tolerance test; SNP – single nucleotide polymorphism (DOCX) [file pone.0034035.s003.docx]

**Table S2. Associations of *SERPINF1* SNPs with glycaemia and insulin sensitivity (overall cohort and clamp subgroup)**

|  | Genotype | N  overall cohort | Fasting glucose (mmol/L) | Glucose 120 min OGTT (mmol/L) | Fasting insulin (pmol/L) | HOMA-IR (mmol*mU*L^-2^) | ISI OGTT  (*10^15^ L^2^*mol^-2^) | N  clamp subgroup | ISI clamp  (*10^6^ L*kg^-1^*min^-1^) |
| --- | --- | --- | --- | --- | --- | --- | --- | --- | --- |
| rs11658342 | GG | 784 | 5.14 ±0.54 | 6.38 ±1.64 | 68 ±59 | 2.68 ±2.51 | 15.6 ±10.7 | 203 | 0.085 ±0.057 |
|  | GA | 909 | 5.14 ±0.56 | 6.35 ±1.64 | 74 ±64 | 2.93 ±2.72 | 14.7 ±10.3 | 216 | 0.082 ±0.054 |
|  | AA | 278 | 5.16 ±0.55 | 6.31 ±1.70 | 70 ±58 | 2.75 ±2.44 | 14.9 ±9.7 | 65 | 0.087 ±0.058 |
| p_add_/p_dom_ | - | - | 0.6/0.4 | 0.05/0.06 | 0.4/0.2 | 0.5/0.3 | 0.4/0.3 | - | 0.4/0.3 |
|  | | | | | | | | | |
| rs1136287 | TT | 788 | 5.16 ±0.55 | 6.38 ±1.69 | 73 ±62 | 2.85 ±2.57 | 14.9 ±10.4 | 182 | 0.084 ±0.064 |
|  | TC | 953 | 5.13 ±0.55 | 6.33 ±1.62 | 71 ±60 | 2.78 ±2.62 | 15.0 ±10.1 | 244 | 0.082 ±0.044 |
|  | CC | 233 | 5.13 ±0.57 | 6.40 ±1.60 | 69 ±61 | 2.71 ±2.63 | 16.1 ±11.6 | 60 | 0.095 ±0.068 |
| p_add_/p_dom_ | - | - | 0.6/0.8 | **0.0279**/0.09 | 0.6/0.5 | 0.5/0.5 | 0.4/0.4 | - | 0.7/0.7 |
|  | | | | | | | | | |
| rs12603825 | GG | 1,039 | 5.14 ±0.57 | 6.32 ±1.63 | 71 ±61 | 2.80 ±2.65 | 15.2 ±10.4 | 258 | 0.088 ±0.054 |
|  | GA | 784 | 5.14 ±0.53 | 6.40 ±1.67 | 72 ±61 | 2.82 ±2.54 | 14.9 ±10.3 | 191 | 0.078 ±0.054 |
|  | AA | 144 | 5.18 ±0.55 | 6.43 ±1.61 | 70 ±63 | 2.77 ±2.62 | 15.4 ±10.7 | 34 | 0.092 ±0.070 |
| p_add_/p_dom_ | - | - | 0.8/1.0 | 1.0/0.9 | 0.2/0.1 | 0.3/0.2 | 0.1/0.08 | **-** | 0.6/0.8 |
|  | | | | | | | | | |
| rs2071021 | AA | 937 | 5.15 ±0.55 | 6.39 ±1.69 | 72 ±60 | 2.82 ±2.49 | 14.8 ±10.2 | 217 | 0.081 ±0.060 |
|  | AG | 861 | 5.15 ±0.56 | 6.32 ±1.62 | 71 ±62 | 2.82 ±2.71 | 15.2 ±10.4 | 224 | 0.085 ±0.048 |
|  | GG | 176 | 5.08 ±0.54 | 6.39 ±1.54 | 66 ±61 | 2.59 ±2.66 | 16.3 ±11.6 | 45 | 0.095 ±0.067 |
| p_add_/p_dom_ | - | - | 0.9/0.9 | 0.2/0.4 | 0.9/0.5 | 0.9/0.5 | 0.9/0.8 | **-** | 0.1/0.2 |
|  | | | | | | | | | |
| rs6828 | CC | 1,005 | 5.13 ±0.55 | 6.33 ±1.60 | 71 ±61 | 2.80 ±2.68 | 15.1 ±10.4 | 257 | 0.085 ±0.053 |
|  | CT | 806 | 5.15 ±0.55 | 6.38 ±1.69 | 72 ±61 | 2.81 ±2.52 | 15.2 ±10.5 | 189 | 0.084 ±0.058 |
|  | TT | 161 | 5.18 ±0.56 | 6.44 ±1.66 | 72 ±60 | 2.82 ±2.52 | 14.3 ±9.3 | 40 | 0.080 ±0.057 |
| p_add_/p_dom_ | - | - | 0.2/0.2 | 0.9/1.0 | 0.6/0.5 | 0.8/0.7 | 0.6/0.3 | - | 0.5/0.4 |

Data are shown as unadjusted raw data (means ±SD). Prior to statistical analysis, all parameters were adjusted for gender, age, and bioelectrical impedance-derived percentage of body fat. p_add_ – p-value, additive inheritance model; p_dom_ – p-value, dominant inheritance model; nominal associations are marked by bold fonts. HOMA-IR – homeostasis model assessment of insulin resistance; ISI – insulin sensitivity index; OGTT – oral glucose tolerance test; SNP – single nucleotide polymorphism
